# Evaluating quality of life tools in North American patients with erythropoietic protoporphyria and X‐linked protoporphyria

**DOI:** 10.1002/jmd2.12052

**Published:** 2019-09-14

**Authors:** Hetanshi Naik, Jessica R. Overbey, Robert J. Desnick, Karl E. Anderson, D. Montgomery Bissell, Joseph Bloomer, Herbert L. Bonkovsky, John D. Phillips, Bruce Wang, Ashwani Singal, Manisha Balwani

**Affiliations:** ^1^ Department of Genetics and Genomic Sciences Icahn School of Medicine at Mount Sinai New York New York; ^2^ Department of Population Health Science and Policy Icahn School of Medicine at Mount Sinai New York New York; ^3^ Department of Preventive Medicine and Community Health University of Texas Medical Branch Galveston Texas; ^4^ Department of Medicine and Liver Center University of California San Francisco California; ^5^ Department of Medicine University of Alabama Birmingham Alabama; ^6^ Department of Medicine (Section on Gastroenterology & Hepatology) Wake Forest NC Baptist Medical Center Winston‐Salem North Carolina; ^7^ Department of Internal Medicine University of Utah Salt Lake City Utah

**Keywords:** erythropoietic proto, porphyria, PROMIS, quality of life

## Abstract

**Background:**

Erythropoietic protoporphyria (EPP) and X‐linked Protoporphyria (XLP) are rare photodermatoses presenting with severe phototoxicity. Although anecdotally, providers who treat EPP patients acknowledge their life‐altering effects, tools that fully capture their impact on quality of life (QoL) are lacking.

**Methods:**

Adult patients with EPP/XLP were given four validated QoL tools: the Patient Reported Outcomes Measurement Information System 57 (PROMIS‐57), the Hospital Anxiety and Depression Scale (HADS), the Illness Perception Questionnaire Revised (IPQR), and an EPP‐Specific tool. All patients received the PROMIS‐57 while the HADS, IPQR, and EPP‐Specific tools were introduced at a later date. Associations between responses and clinical phenotypes were explored.

**Results:**

Two hundred and two patients were included; 193 completed PROMIS‐57, 104 completed IPQR, 103 completed HADS, and 107 completed the EPP‐Specific tool. The IPQR showed that patients strongly believed EPP/XLP had a negative impact on their lives. Mean scores in anxiety and depression domains of both HADS and PROMIS‐57 were normal; however, anxiety scores from HADS were borderline/abnormal in 20% of patients. The EPP‐Specific tool revealed a decreased QoL in most patients. The PROMIS‐57 showed that 21.8% of patients have clinically significant pain interference. Several tool domains correlated with measures of disease severity, most being from the PROMIS‐57.

**Conclusions:**

Impaired QoL is an important consequence of EPP/XLP. PROMIS‐57 was most sensitive in evaluating impaired QoL in EPP/XLP. Further research is needed to compare the effectiveness of it for assessing response to treatment.

**SYNOPSIS**

Quality of life in erythropoietic protoporphyria is not well described as previous studies used dermatologic or treatment specific tools, this is the first robust assessment of QoL tools, which found that ~20% of patients have significant pain interference, and the PROMIS‐57 is the most sensitive to disease severity.

## 1 INTRODUCTION

Erythropoietic protoporphyria (EPP) and X‐linked protoporphyria (XLP) are rare, inherited photodermatoses that result in overproduction of erythrocyte protoporphyrin‐IX (ePPIX). They present with childhood‐onset, severe, painful phototoxicity.^1^, ^2^, ^3^

Sun‐exposed skin, typically on the face and dorsum of the hands, reacts with tingling, burning, and/or itching that may progress rapidly to severe pain, erythema, and swelling.^4^ Severity is variable, but most patients can only tolerate <30 minutes of sun exposure.^2^, ^5^, ^6^ The pain can be excruciating, does not respond well to analgesics, including opioids, and may last for days.^2^, ^4^ The disease necessitates avoiding sunlight as much as possible, which greatly interferes with daily life. As visible blistering is atypical, and chronic skin changes develop slowly or may be overlooked or absent, the diagnosis is often delayed. Management is limited to sun‐protection, as there are currently no FDA‐approved therapies. Due to these severe phototoxic reactions, patients typically develop a conditioned behavior to avoid sunlight, which greatly limits their daily activities.^5^, ^7^ However, effects on quality of life (QoL) are not well described, and the tools most effective for assessing this are not defined.

The dermatology life quality index (DLQI), which has been validated for conditions like eczema and vitiligo,^5^, ^8^ was previously used to assess 176 adults with EPP, and showed that 70% of patients indicated EPP had a large effect on their life in the preceding week.^5^ Additionally, porphyrin levels and time to onset of symptoms were correlated with DLQI scores.^5^

An EPP‐specific QoL tool was developed for clinical trials evaluating afamelanotide, a synthetic analog of α‐melanocyte stimulating hormone. It demonstrated increased pain‐free sun exposure and improved QoL in adults with EPP.^9^ The DLQI was also used and was uninformative when measuring treatment response.^9^

Although the DLQI and EPP‐QoL have been the main tools used to assess QoL in EPP, they have significant limitations. The DLQI was designed for dermatologic conditions with both visible skin lesions and effective treatment. The EPP‐Specific QoL tool asks questions about how the disease affects daily activities (attending school/work, interference with outdoor activities, and so on), and interprets most questions as a “disease severity” domain. Neither specifically assesses the severe pain and conditioned response to avoid sunlight, and how this impacts QoL. Having a robust QoL tool for this population will be important not only for accurately characterizing EPP patients' well‐being, but also for assessing the efficacy of potential new therapies. To address this we evaluated four tools in a large US cohort of adult EPP patients to identify which is most sensitive to disease specific QoL issues.

## 2 MATERIALS AND METHODS

The studies (NCT01561157 and NCT01688895) were performed at six sites of the Porphyrias Consortium of the Rare Diseases Clinical Research Network and conducted in accord with the Declaration of Helsinki. Each site's Institutional Review Board approved the study and informed consent was obtained. Study procedures were described previously.^6^ All patients included had a confirmed diagnosis of EPP with significantly elevated ePPIX. Patients ≥18 years old were given paper copies of the Patient Reported Outcomes Measurement Information System 57 (PROMIS‐57) Version 1 scale,^10^ and a sub‐set also completed, in no specific order, the Hospital Anxiety and Depression Scale (HADS),^11^ the Illness Perception Questionnaire Revised (IPQR),^12^ and a modified EPP‐Specific QoL tool. A protocol modification to add the HADS, IPQR, and EPP‐Specific tools was done after study initiation, therefore not all subjects received all tools. The order in which the tools was given was not randomized. These tools, including PROMIS‐57, were chosen after careful review of the domains they capture and discussion among the investigators regarding what are relevant issues for EPP patients, and gaps in current knowledge. The recall period of 2 months for the EPP‐Specific tool was changed to 1 week to align with the other tools to allow for better comparison, and because investigators felt the longer time period might generate too much variability,^13^, ^14^, ^15^ as EPP symptoms are affected by weather and season, 2 months captures a long length of time where these may change. Table 1 lists the tools, what they measure, briefly describes the scoring methods, and interpretation of scores. The HADS was modified to include a single open‐ended free text question asking if patients would like to share anything else, responses to this were analyzed thematically.^16^

**Table 1:** Tools evaluated

Tool	Measures	Domains	Scoring method	Score interpretation
*Illness perception questionnaire‐revised*	An individual's beliefs and feelings about an illness	Cause (of the illness) timeline of the illness (general) Timeline‐cyclical consequences (of the illness) Personal control treatment control Emotional representations	Each item was scored from 1 to 5 on a Likert scale. Items within each domain were totaled for final domain scores.	No normal/reference ranges. High scores on the identity, timeline, consequences, and cyclical dimensions represent strongly held beliefs about the number of symptoms attributed to the illness, the chronicity of the condition, the negative consequences of the illness, and the cyclical nature of the condition. High scores on the personal control, treatment control, and coherence dimensions, represent positive beliefs about the controllability of the illness and a personal understanding of the condition
*Hospital anxiety and depression scale*	Anxiety and depression	Anxiety Depression	Each item was scored from 0 to 3 on a Likert scale. Items within each domain were totaled for final domain scores.	0‐7 normal 8‐10 borderline abnormal 11‐21 abnormal
*Modified EPP‐specific QoL*	Disease severity and overall QoL/wellbeing	Disease severity (S) QoL/wellbeing (Q)	Each item was scored from 0 to 3 on a Likert scale. Items within each domain were totaled and transformed onto a 0‐100 scale	No normal/reference ranges. Higher scores for the S domain reflect lower severity, and higher satisfaction/QoL for the Q domain
*PROMIS‐57*	Social, mental, and physical health	Physical function Anxiety Depression Fatigue Sleep disturbance Satisfaction with social role Pain interference	Each item was scored from 1 to 5 on a Likert scale. Items within each domain were totaled. Raw scores converted to standardized T scores by PROMIS Assessment Center	General population mean standardized score is 50 for each domain, with a SD of 10. Higher scores in each domain indicate more of that domain.

## 3 STATISTICAL ANALYSIS

All tools were scored according to established methods^10^, ^11^, ^12^ and the modified EPP‐Specific tool was scored as previously reported^9^ Differences in scores were assessed by sex, disease (EPP vs XLP), reported medical diagnosis of depression and/or anxiety (combined), either ongoing or in the past, and history of abnormal liver enzymes (patient‐reported history or abnormal serum aminotransferase levels at enrollment) using two‐sample *t*‐tests. Associations among scores and clinical features assessing disease severity (time to first symptom, recovery time, ePPIX levels, and so on) were explored using Spearman's or Pearson's correlation coefficients as appropriate. These were selected as measures of disease severity because investigators determined they were important, or patients reported them as issues of concern. Analyses were considered exploratory and conducted at the 0.05 significance level using SAS version 9.4 (SAS, Cary, North Carolina).

## 4 RESULTS

The cohort included 202 adults with a mean age at enrollment of 40.9 years (SD 14.5, range 18‐77), and 180 (89.1%) EPP and 22 (10.9%) XLP patients. There were approximately equal numbers of males and females of both EPP and XLP patients; 94 EPP males and 86 females, and 10 XLP males and 12 females. Mean age at onset of symptoms was 4.4 years (SD 4.4; Table 2). Comparisons of responses for each tool between sex (males vs females) and disease (EPP vs XLP) were unremarkable. Characteristics of this cohort have been previously reported, however only the portion who completed these tools is reported here.^6^ Of the 202 patients, 104 completed the IPQR, 103 completed the HADS, 107 completed the EPP‐Specific tool, and 193 completed the PROMIS‐57; 101 patients completed all of the tools. The demographics and disease characteristics of patients completing all tools was similar to the overall cohort, with the exception of the proportion of patients with a history of liver enzyme abnormalities (23.5% vs 31.9% for those who completed all tools vs the total cohort).

**Table 2:** Characteristics of patient sample

	N = 202	%
EPP	180	89.1
Male	94	46.5
Female	86	42.5
XLP	22	10.9
Male	10	4.9
Female	12	5.9
Age at enrollment	Mean 40.9, SD 14.5	
Age at onset of symptoms	Mean 4.4, SD 4.4	
Time to first symptom	N = 191	
<10 min	58	28.7
11‐30 min	69	34.2
31‐60 min	28	13.9
1‐3 h	30	14.9
>3 h	6	3.0
Unknown/not reported	11	5.4
Recovery time	N = 195	
4‐24 h	6	3.0
1‐3 days	119	58.9
4‐7 days	62	30.7
>7 days	8	4.0
Unknown/not reported	7	3.5
Number of phototoxic episodes in past year	N = 194	
1‐2	44	21.8
3‐10	90	44.6
11‐30	39	19.3
31‐60	8	4.0
>60	13	6.4
Unknown/not reported	8	4.0
History of abnormal liver enzymes N = 191	61	31.9
History of depression and/or anxiety	39	19.3

### 4.1 Descriptive statistics and summary scores

#### 4.1.1 Illness Perception Questionnaire‐Revised

The IPQR was completed by 104 patients (96 EPP, 8 XLP; Table 3). The higher scores (>20) on the timeline and consequences domains reflect overall strong beliefs that the disease is chronic and has a negative impact. The moderate cyclical domain scores indicate that the disease is viewed as persistent rather than cyclic. Control scores indicate that patients believed they understood their disease and had personal control over it (high coherence and personal control domain scores), but felt that effective treatment was lacking (low treatment control scores, Table 2). High emotional representation scores reflect that the disease negatively affects patients' mood.

**Table 3:** IPQR, HADS, modified EPP‐specific QoL, and PROMIS scores

Domains			N	Score			
				Mean	SD	Range	Maximum possible
IPQR^a^							
Timeline			104	23.4	2.2	13‐25	25
Consequences				23.2	4.3	12‐30	30
Personal control				19.0	4.9	9‐28	30
Treatment control				9.2	2.8	2‐15	15
Illness coherence				19.0	4.2	7‐25	25
Timeline‐cyclical				10.5	3.8	4‐20	20
Emotional representations				18.8	5.5	6‐30	30
HADS							
Anxiety			103	4.6	4.1	0‐19	21
Depression				1.9	2.1	0‐10	
EPP‐specific QoL							
S domain			107	58.9	32.6	0‐100	100
Q domain				30.8	27.4	0‐100	
Total score				54.3	30.0	0‐100	

Domains	N	Score			Number of patients with >1 SD impairment (%)		Maximum possible
		Mean	SD	Range			
Adult PROMIS							
Pain interference	193	50.5	10.3	40‐77	42 (21.8)		100
Depression	192	44.9	8.7	38‐72	13 (6.8)		
Anxiety	192	47.4	10.3	37‐83	23 (12.0)		
Fatigue	193	48.2	11.1	33‐78	28 (14.5)		
Physical function	192	51.9	8.3	20‐59	16 (8.3)		
Sleep disturbance	193	49.5	8.9	30‐74	20 (10.4)		
Satisfaction with social role	188	53.5	10.2	27‐66	15 (8.0)		

a A modified version without the identity component was used as it was not applicable in EPP.

#### 4.1.2 Hospital Anxiety and Depression Scale

The HADS was completed by 103 patients (95 EPP, 8 XLP), and mean scores for anxiety and depression were within the normal range (Table 3); however, 20% (21/103) of patients had borderline or abnormal anxiety levels, and 9.7% (10/103) had borderline or abnormal depression levels.

When asked “feel free to make any additional comments about how your illness has affected your quality of life” at the end of the HADS, 49 patients responded. Open responses revealed that many patients thought about what their lives could have been without EPP, and how their symptoms significantly impacted them:

“EPP has made my life very lonely and isolated…If there is an occasion where I must go in the sun…I become very apprehensive and agitated”

“I am not who I could have been.”

“As a child EPP was "torture" in my mind. The rashes, scars & sun avoidance were extreme issues in my life…The stigma of being a "freak" was an emotional toll. As an adult, I learned to accept the EPP.”

#### 4.1.3 Modified EPP‐Specific QoL

Higher scores for the S domain reflect lower severity, and for the Q domain higher satisfaction/QoL. Mean scores of the 107 patients who completed this tool are shown in Table 3. There are no predetermined cut‐offs for this tool to specify low vs high QoL. However, the mean score of 30.8 on the Q domain, well below the maximum score of 100, likely indicates decreased QoL. Cronbach's alpha was 0.95, showing good internal consistency of this modified tool.

#### 4.1.4 PROMIS‐57

Of the 202 patients, 193 adults completed the PROMIS‐57, mean scores for each domain were similar to the general population (Table 3). A higher PROMIS score represents more of the domain being measured. For example higher anxiety scores mean greater anxiety, where a score of 60 is one SD worse than the general population average of 50, while higher physical function scores mean better physical functioning. The percentage of scores indicating >1 SD of impairment, which can be considered clinically significant, are shown in Table 2; notably 21.8% (42/193) of patients have clinically significant pain interference.

### 4.2 Associations of QoL tool scores with disease severity

Table 2 shows the characteristics used to define disease severity for this cohort. Disease severity was variable for this cohort with a wide range of patient‐reported time to first symptom. Most patients reported recovering from symptoms within 1 to 3 days (61.0%), 31.9% reported a history of abnormal liver enzymes or had abnormal results at baseline, and 19.3% reported a diagnosis of anxiety and/or depression. Of those with a diagnosis of anxiety and/or depression, only four patients reported that this was not an ongoing issue (4/39).

#### 4.2.1 Illness Perception Questionnaire Revised

A history of liver enzyme abnormalities did not significantly affect IPQR scores. Mean scores for the personal control, treatment control, and emotional representations domains were significantly worse for patients with a history of anxiety and/or depression (Personal control: positive history mean 16.8 ± 5.0 vs negative history mean 19.5 ± 4.8 [*P* = .03]; Treatment control: positive history mean 7.6 ± 3.2 vs negative history mean 9.5 ± 2.6 [*P* = .009]; Emotional representations: positive history mean 22.8 ± 4.0 vs negative history mean 17.9 ± 5.3 [*P* = .0004]). The consequences, treatment control, and emotional representations domains also had significant correlations with scores being higher in those with shorter time to first symptoms (Spearman *r* = −0.38, *P*‐value = .0001; Spearman *r* = 0.20, *P*‐value = .04; and Spearman *r* = −0.30, *P*‐value = .003, respectively). In addition, the consequences, timeline‐cyclical, and emotional representations domains had significant correlations with scores being higher in those with longer recovery time (Spearman *r* = 0.29, *P*‐value = .003; Spearman *r* = 0.21, *P*‐value = .04; and Spearman r = 0.20, P‐value = .05, respectively). None of the domain scores were significantly correlated with number of phototoxic episodes in the past year, baseline ePPIX levels, or age at onset of symptoms.

#### 4.2.2 Hospital Anxiety and Depression Scale

Mean scores for anxiety and depression were not significantly different between those with a history of liver enzyme abnormalities and those without. A medical history of anxiety and/or depression did affect mean scores for the anxiety domain (positive history of anxiety mean 7.9 ± 5.2 vs negative history mean 3.9 ± 3.5 [*P* = .01]), however depression scores were not significantly different. Anxiety and depression scores did not correlate with time to first symptom, but did correlate with recovery time (anxiety domain Spearman *r* = 0.20, *P*‐value = .05; depression domain Spearman *r* = 0.29, *P*‐value = .003). The anxiety domain also had a significant correlation with number of phototoxic episodes (Spearman *r* = 0.21, *P*‐value = .04). Scores were not significantly correlated with ePPIX or age at onset of symptoms.

#### 4.2.3 Modified EPP‐specific QoL

Neither a history of liver enzyme abnormalities nor anxiety and/or depression affected mean scores for the modified EPP‐Specific QoL domains. But the severity domain was significantly correlated with time to first symptom (Spearman *r* = 0.47, *P*‐value<.0001). Neither of the domains was significantly associated with recovery time, number of phototoxic episodes, ePPIX, or age at onset.

#### 4.2.4 PROMIS‐57

**Figure 1:**
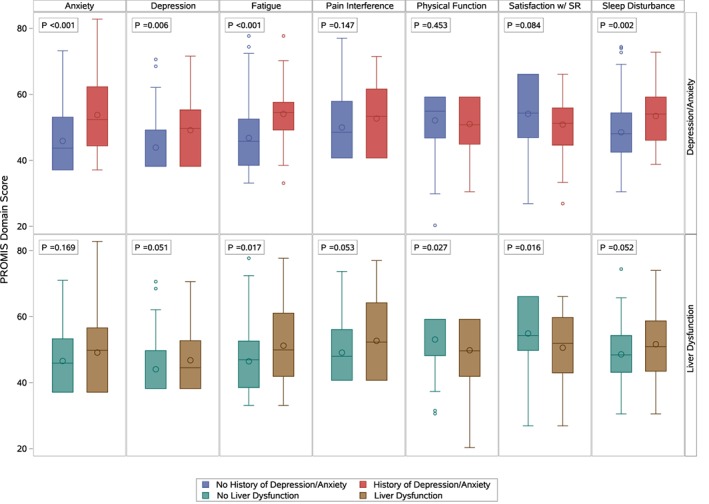
EPP. PROMIS scores and history of anxiety/depression and liver enzyme abnormalities. Box plots of PROMIS scores across seven domains by history of anxiety and/or depression is shown on the top row, and by history of liver enzyme abnormalities is shown on the bottom row. For each domain, a higher PROMIS score represents more of the domain being measured. In each plot, the small circle inside the box indicates the sample mean. The line inside the box is the median or 50th percentile. The bottom line of the box is the 1st quartile or 25th percentile and the top line is the 3rd quartile or 75th percentile. The bottom “whisker” is the minimum observation within the lower fence and the top “whisker” is the maximum observation within the upper fence. Lower fence is computed by Q1‐1.5* (IQR; interquartile range) and upper fence is computed by Q3 + 1.5*(IQR). Circle markers outside of the fences are defined as outliers

Mean scores for physical function, fatigue, and satisfaction with social role were significantly different between those with a history of liver enzyme abnormalities and those without (Figure 1). Pain interference, physical function, fatigue, and satisfaction with social role scores were significantly different by time to symptom onset after sun exposure (Table 4). Pain interference and sleep disturbance were also significantly associated with recovery time (Spearman *r* = 0.18, *P*‐values .01 and Spearman *r* = 0.18, *P*‐value .02, respectively). Pain interference was the only domain significantly associated with the number of phototoxic episodes (Spearman *r* = 0.17, *P*‐value .02). Those who reported a history of anxiety and/or depression had significantly worse mean scores for depression, fatigue, anxiety, and sleep disturbance (Figure 1). Pain Interference scores significantly correlated with ePPIX (Pearson *r* = 0.17, *P*‐value = .03). No correlations were observed with age at onset.

**Table 4:** PROMIS scores and time to first symptom

Domains	Time to first symptom^a^	N	Mean score	SD	Spearman *r*	*P*‐value
Pain interference	<10 min	57	53.9	10.9	−0.23	0.0016
	11‐30 min	66	50.1	11.1		
	31‐60 min	26	48.6	8.5		
	1‐3 h	28	46.0	7.8		
	>3 h	6	50.0	8.0		
Depression sadness	<10 min	56	44.9	9.2	−0.11	0.14
	11‐30 min	66	46.8	8.3		
	31‐60 min	26	44.1	9.2		
	1‐3 h	28	42.2	7.5		
	>3 h	6	41.7	5.6		
Anxiety fear	<10 min	56	47.1	10.1	−0.04	0.57
	11‐30 min	66	48.7	11.3		
	31‐60 min	26	47.9	9.0		
	1‐3 h	28	44.5	9.0		
	>3 h	6	48.8	7.9		
Fatigue	<10 min	57	49.7	10.2	−0.16	0.03
	11‐30 min	66	49.0	12.0		
	31‐60 min	26	47.5	10.4		
	1‐3 h	28	43.8	11.0		
	>3 h	6	49.5	13.3		
Physical function	<10 min	56	49.0	10.1	0.25	0.0006
	11‐30 min	66	51.7	7.3		
	31‐60 min	26	53.6	6.1		
	1‐3 h	28	55.4	7.0		
	>3 h	6	54.7	7.9		
Sleep disturbance	<10 min	57	49.3	9.2	−0.07	0.35
	11‐30 min	66	51.9	8.5		
	31‐60 min	26	49.5	7.0		
	1‐3 h	28	46.6	8.8		
	>3 h	6	47.4	9.6		
Satisfaction with social roles	<10 min	54	51.0	10.8	0.22	0.003
	11‐30 min	65	52.4	10.2		
	31‐60 min	25	57.3	7.1		
	1‐3 h	28	56.3	10.3		
	>3 h	6	56.6	11.2		

a Subjects who did not report their time to first symptoms were not included.

## 5 DISCUSSION

EPP has a large impact on patient QoL. Our study examines four validated QoL tools to determine which best captures this. Previous studies in Europe used standard dermatologic tools, which are limited as they do not assess all patient‐reported outcomes for EPP. EPP has unique symptoms and implications for patients' lives. The only disease specific tool for EPP was created for use in clinical trials assessing afamelanotide,^9^ and validation studies have not been published. A tool that is sensitive to all aspects of the disease is lacking.

Clinical features in our cohort, such as age at onset and time to first symptom, were consistent with previous reports.^5^, ^17^ The IPQR showed that patients believe their disease has a large negative impact on their lives, but that they have some control in dealing with it. Understanding patients' perceptions is critical to providing appropriate counseling; however, it is unlikely that these perceptions will change and administering this tool longitudinally may not be clinically useful.

HADS scores revealed that ~20% of patients with EPP had borderline or abnormal scores on the anxiety domain, and ~10% had borderline or abnormal scores on the depression domain. In the United States 8.1% of adults over 20 had depression, and the estimated lifetime prevalence of any anxiety disorder is ~15%.^18^ Levels of anxiety and depression for our cohort were not significantly different from general population estimates. As well, the depression domain score was not significantly associated with a history of anxiety and/or depression, which is surprising. It is possible the depression domain was not sensitive enough for this cohort, or combining a history of anxiety with depression affected these results. As only four patients had a history of anxiety/depression that was in the past, it is unlikely this affected the scores. Added free text responses in the HADS; however, illustrated that patients struggle with isolation caused by EPP, and many felt the disease was particularly difficult to manage in childhood. Therefore, disease impact may be more significant in children, and as adults they have learned to cope.

The modified EPP‐specific tool showed decreased QoL in these patients, which is consistent with previous reports using the original tool.^5^, ^9^ However, this is the first study to use the tool in a non‐treatment setting. While we cannot exclude the possibility that modifying the recall time affected the sensitivity of this tool, it is also possible that the tool is not ideal for monitoring general QoL without a treatment.

Several of the PROMIS‐57 domains had a relatively large proportion of patients with >1 SD impairment, similar to what has been observed when using these scales in other chronic diseases.^19^ The pain interference domain had a particularly large proportion of patients with clinically significant impairment. The PROMIS scales are considered psychometrically robust and have been validated in several diseases,^19^, ^20^, ^21^ but have not been used in many rare diseases. Some questions may not be useful for EPP and may be limiting the observed impact of their domains. As well, additional studies are needed to determine if this tool is sensitive to treatment response.

All the tool scores correlated with measures of disease severity, some more so than others; however, the PROMIS domains appeared to be the most sensitive. Several PROMIS domains had significantly different scores by disease severity measures, specifically the pain interference, physical function, and satisfaction with social roles domains. The sensitivity of this tool should be further assessed to determine if the PROMIS domains can be used to track disease progression over time. EPP pain is generally only considered an issue during phototoxic reactions^2^; however, the pain interference domain was significantly associated with several markers of disease severity. This is striking as generally the tools were administered while patients were in clinic and not reporting active phototoxic reactions. Pain interference may be more important to daily functioning than originally suspected, even in patients who are adept at avoiding sun exposure.

It is possible the PROMIS domains were identified as most sensitive due to the larger sample size; however, the representation of patients with various measures of disease severity were varied enough in those who completed the HADS, IPQR, and EPP‐Specific to observe meaningful differences in scores. In fact several of the other tools' domains did associate with disease severity measures. However, the PROMIS domains are likely most sensitive because they cover a more varied set of domains than the other tools.

There are several factors which impact the accuracy of measuring QoL in EPP patients. The tools compared in this study use a 7‐day recall, but wide variations in weather and amount of time that can be spent in the sun may affect results, and a longer recall period may be more appropriate for this disease. However, accuracy is likely to decrease were a longer recall period used.

Seasonality should also be considered, especially in the context of measuring a treatment response. The EPP‐Specific QoL tool was analyzed for whether the scores varied with the season during afamelanotide treatment.^22^ The mean scores during winter months were higher than during summer, suggesting better QoL in winter.^22^

Anecdotally, many patients have expressed to the investigators that they struggle with answering these tools as they have learned to cope with their disease over time, becoming experts at avoiding situations, which may put them at risk of developing phototoxic reactions. This is not captured as an impact in existing tools, despite the fact that it greatly affects patients' lives. We believe this leads to significant under‐reporting of the impact of EPP. Due to the nature of this disease and lack of effective treatments, patients do not experience what is “normal” or “improved,” but only compare periods in their lives with and without efforts to avoid sunlight.

EPP is the most common type of porphyria in children^2^; many adults described struggling with the disease and being depressed as children in the open response questions, and found it particularly difficult to navigate school life.^23^ Therefore, it is critical to develop tools for assessing QoL effects in children with EPP. Using such tool in clinical practice can allow for early intervention, such as referring to psychology, social work, or other services to help patients cope, if responses show a decreased QoL score over time.

The analyses assessing correlations with disease severity were limited by the smaller number of patients who completed the IPQR, HADS, and modified EPP‐Specific tools compared to the PROMIS; however, results are still clinically useful in assessing these tools. As well, seasonality and geographic location were not accounted for in this study and should be in future evaluations of QoL tools in this population.

In conclusion, we compared four QoL tools in adult EPP patients and determined that overall the PROMIS‐57 is most sensitive to disease severity. While this tool may be clinically useful when evaluating patients, it may require supplementation with disease‐specific questions. An ideal QoL tool would be both clinically useful and effectively measure treatment response. A major goal of treatment is to enable patients to spend more time in the sun, and freedom to do this would greatly improve their QoL. Therefore measurement of QoL has a central role in documenting improvement in EPP.

## AUTHOR CONTRIBUTIONS

H.N. and M.B. had full access to all of the data in the study and take responsibility for the integrity of the data and the accuracy of the data analysis. Study concept and design: K.E.A., M.B., D.M.B., H.L.B., J.B., J.D.P., R.J.D., H.N. Acquisition, analysis, and interpretation of data: M.B., J.B., K.E.A., D.M.B., H.L.B., J.D.P., R.J.D., H.N., J.R.O., B.W., A.S. Drafting of the manuscript: H.N., M.B. Critical revision of the manuscript for important intellectual content: M.B., J.B., K.E.A., D.M.B., H.L.B., J.D.P., R.J.D., H.N., J.R.O., B.W., A.S. Statistical analysis: J.R.O., H.N. Obtained funding: J.B., K.E.A., D.M.B., H.L.B., J.D.P., R.J.D., M.B. Administrative, technical, or material support: R.J.D., H.N. Study supervision: M.B., R.J.D.

## CONFLICT OF INTEREST

Hetanshi Naik reports consultancies with Alnylam. Jessica R. Overbey, Bruce Wang, Ashwani Singal, and Joseph Bloomer declare no conflict of interest. Robert J. Desnick reports consultancies from Alnylam Pharmaceuticals, Recordati Rare Diseases, and Mitsubishi Tanabe. Karl E. Anderson reports consultancies from Alnylam and grant (clinical trial) support from Alnylam and Mitsubishi Tanabe. D. Montgomery Bissell reports honoraria from Recordati and grant (clinical trial) support from Alnylam and Mitsubishi Tanabe. Herbert L. Bonkovsky reports consultancies form Alnylam, and Recordati Rare diseases and grant (clinical trial) support from Alnylam and Mitsubishi Tanabe. John D. Phillips reports consultancies from Alnylam, Recordati, and Agios and grant (clinical trial) support from Mitsubishi Tanabe. Manisha Balwani reports consultancies Alnylam, Recordati, and Mitsubishi Tanabe, and grant (clinical trial) support from Alnylam and Mitsubishi Tanabe.

## INFORMED CONSENT

All procedures followed were in accordance with the ethical standards of the responsible committee on human experimentation (institutional and national) and with the Helsinki Declaration of 1975, as revised in 2000, and in 2013. Informed consent was obtained from all patients for being included in the study.

## ETHICS APPROVALS

Each sites' Institutional Review Boards approved these studies and all patients provided written consent.

## ANIMAL RIGHTS

This article does not contain any studies with animal subjects performed by the any of the authors.

## FUNDING INFORMATION

This research was supported in part by the Porphyrias Consortium (U54DK083909), which is a part of the NCATS Rare Diseases Clinical Research Network (RDCRN). RDCRN is an initiative of the Office of Rare Diseases Research (ORDR), NCATS, funded through a collaboration between NCATS and the NIDDK. MB is the recipient of a NIH Career Development Award (K23 DK095946). B.W. and A.S. are supported in part by the American Porphyria Foundation's “Protect the Future Program.”
